# Medical Cannabis Use Among Canadian Veterans and Non-Veterans: A National Survey

**DOI:** 10.1089/imr.2023.0022

**Published:** 2023-10-12

**Authors:** Gunel Valikhanova, Yuka Kato, Mary-Ann Fitzcharles, Mark Ware, Deborah Da Costa, Ilka Lowensteyn, Ho Sum Cheung, Steven Grover

**Affiliations:** ^1^ Department of Medicine, McGill University, Montreal, Canada.; ^2^ Department of Mathematics & Statistics, Concordia University, Montreal, Canada.; ^3^ Department of Rheumatology, McGill University, Montreal, Canada.; ^4^ Alan Edwards Pain Management Unit, McGill University, Montreal, Canada.; ^5^ Department of Family Medicine, McGill University, Montreal, Canada.; ^6^ Division of Clinical Epidemiology, McGill University, Montreal, Canada.; ^7^ McGill Comprehensive Health Improvement Program, Faculty of Medicine, McGill University, Montréal, Canada.

**Keywords:** medical cannabis, Veterans, chronic pain, mental health problems

## Abstract

**Background:**

Medical cannabis (MC) is used by Canadian Veterans to manage a wide range of health issues. However, there is little information comparing the reasons for MC use and its perceived effectiveness between Veterans and non-Veterans.

**Objects:**

We compared MC use among a convenience sample of Canadian Veterans and with non-Veteran controls, including demographics, reasons and patterns of use, and perceived effectiveness.

**Methods:**

Between November and December 2021, Canadian Veterans using cannabis were invited to participate in a survey using a national press release, social media, and announcements on online platform dedicated to promoting health among Canadian Veterans and non-Veterans during the pandemic (www.MissionVav.com). The survey was also mentioned in a monthly newsletter from Veteran Affairs Canada. Self-reported effectiveness was evaluated using a 0 to 10 visual analogue scale (0 being not all effective, 10 being the most effective).

**Results:**

The survey was completed by 157 people, including 108 (69%) males and 49 (31%) females. The mean age was 57 years (range 19 to 84). Among responders, 90 (63%) identified as Veterans. The most common reasons for MC use among Veterans included: insomnia (80%), anxiety (73%), and depression (52%). Veterans reported medical conditions such as chronic pain (88%) and arthritis (51%). Compared with non-Veterans, Veterans were significantly more likely to be male (83% vs. 49%), have a higher BMI (35.2 vs. 30.9), to report problems with sleep, anxiety, depression, and PTSD, and to use cannabis in edible form (51% vs. 22%). Self-reported mean effectiveness scores for MC were highest for PTSD (8.4), insomnia (8.2), anxiety (8.1), depression (8.0), and chronic pain (7.6).

**Conclusions:**

We found important differences in user characteristics and cannabis use patterns between Canadian Veterans and non-Veterans. Further controlled studies are required to validate these findings, but these data suggest that orally administered cannabis products may be worth further study.

## Introduction

Cannabis has traditionally been used as a self-remedy for the management of symptoms related to physical illness and psychological health. For many conditions, however, the evidence to support optimal clinical use is of poor quality and/or inconsistent. Canadian Veterans are disproportionately impacted by conditions for which medical cannabis (MC) is frequently used. The Life after Service Study demonstrated that Canadian Veterans, compared with Canadians who have not served, are more prone to suffer from a wide range of physical and mental health issues, including chronic pain, anxiety, post-traumatic stress disorder (PTSD), and insomnia.^1,2^

Among Veterans, annual program expenditures for MC have been increasing rapidly. There are currently 617,800 Veterans in Canada and Veterans Affairs Canada has allocated $153,780,785 for MC to support 18,388 (3%) Veterans in the fiscal year 2021–2022. Program expenditures are expected to reach $195.2 million in the fiscal year 2022/2023.^3,4^

Despite the uncertainty regarding the efficacy of MC, it is apparent that close to 20,000 Veterans, whose cannabis authorizations are paid for by Veterans Affairs Canada, continue to use this product to manage their medical conditions. MC is perceived a low-risk and safe product in comparison to many other drugs.^5,6^ There is, however, clear evidence for the harms associated with cannabis use, including potential adverse effects on mental health and associations with respiratory, cardiovascular, and gastrointestinal events.

A further concern for MC use is the risk of cannabis use disorder (CUD).^7^ CUD reports have grown significantly in the past decade, particularly among Veterans.^8,9^ The perceived net benefit of MC may be, in part, due to the fact that MC is often used to treat multiple conditions in the same individual.^10^ Accordingly, if users believe that they are treating multiple conditions, they may be more willing to accept the risk of an adverse secondary effect.

Little research has been conducted to investigate patterns and perceived effectiveness of MC usage among those who use it regularly. One recently conducted study found that among 9766 older adults using MC, the primary reason was to alleviate pain. A majority of participants reported pain relief (73%), enhanced sleep (65%), and improved mood (53%). Further, 36% reported a reduction in the dosage of opioids.^11^ In another recent study (7362 patients), MC was perceived to significantly reduce anxiety and depression.^12^

Given that Canadian Veterans use MC for a wide range of symptoms, they are an appropriate group to evaluate for further understanding and optimizing the use of MC. They might also help to identify the target conditions that are most likely to respond to MC based on current usage and perceived benefits. It is also essential to understand whether the experience of Veterans is generalizable to other MC users. There has been relatively limited research on this issue, and only one study has explicitly evaluated the differences of the MC usage by Veterans versus non-Veterans, taking into account sex distinctions.^13^

The current study assessed MC use among a convenience sample of Canadians, comparing Veterans and non-Veterans to identify differences in user characteristics (socio-demographic, health, lifestyle etc.) reasons for use, usage patterns, perceived effectiveness, and sex-specific effects.

## Methods

### Recruitment

Between November and December 2021, Canadian MC users, both Veterans and non-Veterans, were recruited to complete an online questionnaire. Respondents were invited to participate in the “CannCorps Study” in a number of ways. Canadian Veterans, family members, and friends were recruited via an online platform dedicated to promoting health among Canadian Veterans and their families (www.MissionVav.com).

The CannCorps Study was highlighted on the MissionVaV landing page, and an active link was provided to the online survey. Also, a posting on social media (“CannCorps” and “MissionVav” Facebook pages) highlighted the survey, which was also mentioned in a monthly newsletter to Veterans from Veteran Affairs Canada. Finally, the general public was invited to participate in a press release to Canadian news agencies.

### Survey

The survey was available in English or French and took <10 min to complete on a computer, tablet, or smartphone. Participants were required to be at least 18 years of age and had used MC at least once in the past month or were considering use in the next month. Anonymity was maintained as no identifiers were collected and the email address of respondents was not recorded. The checklist for Reporting Results of Internet E-Surveys (CHERRIES) has been provided in Supplementary Material.

A delegated review of the study was provided by member(s) of the McGill University Health Centre (MUHC) Research Ethics Board, more precisely its Cells, Tissues, Genetics, and Qualitative research panel. The research project was found to meet scientific and ethical standards for conduct at the MUHC.

The survey questions focused primarily on MC use patterns, reasons for use, and the respondent's perception of efficacy of their specific MC therapy (See Survey Questionnaire in Supplementary Appendix SA1). Sociodemographic data, including age, sex, and ethnicity, were collected. In addition, data were recorded on lifestyle habits that might impact physical and mental health symptoms such as physical activity, consumption of tobacco and alcohol.

Among the 115 questions, 45 (39%) focused on the respondent's perception of effectiveness of MC, and MC use patterns (mode of administration, usage frequency, current daily amount used, preferred dose, usage duration, preferred strain, concentrations of delta-9 tetrahydrocannabinol [THC, and cannabidiol [CBD] etc.). Participants were also questioned about their reasons for MC use, any concerns about MC use, whether they felt that cannabis use was out of control, and how difficult they found it to stop using.

Questions were answered by yes/no, drop-down answer options, multiple-choice responses, open-ended responses, and rating scales. Self-reported effectiveness was evaluated using a 0 to 10 visual analogue scale (0 = not at all effective, 10 = most effective).

### Data analysis

Descriptive statistics were used to summarize all responses. After stratifying the sample by Veteran status, chi-square tests (for categorical variables) and independent *t*-tests (for continuous variables) were used to compare the responses of Veterans versus non-Veterans. Non-parametric tests were used for highly skewed data. Linear and logistic regression models were performed to determine whether cannabis usage among Veterans was significantly different from non-Veterans after adjustment for age and sex. Analyses were also completed for males and females separately. All analyses were conducted using Python version 3.10.

## Results

The survey was completed by 158 respondents. After excluding 10 outliers who provided unrealistic responses, 148 participants were selected for further analyses. Among respondents, 90 (63%) self-identified as Veterans and 58 (37%) as non-Veterans. There were 99 males and 49 females. The mean age was 57 years (range 19 to 84 years), 103 (73%) were married, 128 (82%) identified as Caucasian/White background, and 85 (60%) were retired. Compared with non-Veterans, Veterans were significantly (*p* < 0.05) more likely to be male (83% vs. 49%).

Subsequent analyses were sex specific. Both Veterans and non-Veterans were similar in terms of age, education levels, ethnic origin, and marital status. Both groups reported identical patterns of alcohol and cigarette usage, as well as physical activity habits (Table 1).

**Table 1.** Demographics and Lifestyle Habits of Male and Female Veterans Versus Non-Veterans

	Males (***N*** = 103)		Females (***N*** = 39)	
	Veterans (***N*** = 75)	Non-veterans (***N*** = 22)	Veterans (***N*** = 15)	Non-veterans (***N*** = 24)
Age (mean)	57.2	55.5	52.6	60.7
BMI (mean)	29.9	28.2	26.5	25.8
Background	66 Caucasian/White	20 Caucasian/White	14 Caucasian/White	21 Caucasian/White
	6 Aboriginal	0	1 Aboriginal	2 Latin American
	1 Black	0		
Marital status				
Married	61 (60%)	18 (81%)	12 (80%)	13 (54%)
Divorced	7 (7%)	0	3 (20%)	5 (21%)
Single	7 (7%)	4 (18%)	0	5 (21%)
Education				
Less than high school	2 (2%)	0	1 (7%)	0
High school or equivalent	16 (15%)	3 (14%)	1 (7%)	2 (8%)
University but no degree	14 (13%)	1 (5%)	0	3 (13%)
Technical/community college/CEGEP	33 (32%)	7 (32%)	7 (47%)	7 (29%)
University degree	5 (5%)	6 (27%)	2 (13%)	7 (29%)
Graduate degree	4 (4%)	4 (18%)	0	2 (8%)
Employment				
Employed	15 (14%)	5 (23%)	2 (13%)	6 (25%)
Unemployed	3 (3%)	3 (14%)	3 (20%)	1 (4%)
Retired	50 (49%)	11 (50%)	10 (67%)	14 (58%)
Alcohol consumption				
Never	20 (19%)	3 (14%)	2 (13%)	8 (33%)
2–4 times a month or less	31 (30%)	7 (32%)	6 (40%)	7 (29%)
2–3 times a week	15 (14%)	3 (14%)	6 (40%)	6 (25%)
Daily or almost daily	9 (9%)	9 (41%)	1 (7%)	3 (13%)
Cigarette consumption				
Not at all	66 (64%)	21 (95%)	12 (80%)	24 (100%)
At least once a week	8 (8%)	1 (5%)	0	0
Physical activity				
Walking hours during the past 7 days	4.02 (2)	4.9 (3)	6.8 (2.3)	4.29 (2)
Moderate physical activity (hours)	4.03 (2.2)	4.3 (2)	2.89 (1)	5.9 (3)
Vigorous physical activity (hours)	1.64 (0)	4.37 (0)	1 (0)	1 (0)

Data are presented as *n* (%) or mean (median).

BMI, body mass index.

Male Veterans versus non-Veterans were significantly more likely to report problems with depression (50% vs. 14%, *p* < 0.01), anxiety (72% vs. 18%, *p* < 0.001), and PTSD (36% vs. 9%, *p* < 0.001). Female Veterans vs. female non-Veterans were significantly more likely to use MC for conditions such as PTSD (60% vs. 12%, *p* < 0.001) and arthritis (60% vs. 12%, *p* < 0.001) (Table 2).

**Table 2.** Number and Percentages of Veterans and Non-Veterans Using Medical Cannabis for the Different Conditions

	Males (***N*** = 103)		Females (***N*** = 39)	
	Veterans (***N*** = 75)	Non-veterans (***N*** = 22)	Veterans (***N*** = 15)	Non-veterans (***N*** = 24)
Chronic pain	44 (58.6%)	12 (55%)	12 (80%)	17 (71%)
Headaches/migraines	14 (18.7%)	3 (14%)	6 (40%)	5 (21%)
Acute pain	29 (38.7%)	6 (27%)	6 (40%)	6 (25%)
Sleeping problems	60 (80%)	10 (45%)	12 (80%)	12 (50%)
Muscle spasm	14 (19%)	6 (27%)	6 (40%)	6 (25%)
PTSD	**37 (49%)** ^**^	**1 (5%)** ^**^	**9 (60%)** ^**^	**3 (13%)** ^**^
Depression	**38 (50%)** ^**^	**3 (14%)** ^**^	9 (60%)	6 (25%)
Anxiety	**54 (72%)** ^***^	**4 (18%)** ^***^	12 (80%)	10 (42%)
Arthritis	37 (49%)	6 (27%)	**9 (60%)** ^***^	**3 (12%)** ^***^

The bold values represent all the values that are significantly different.

Data are presented as *n* (%) or mean (median).

^**^ *p* < 0.01, ^***^p < 0.001.

PTSD, post-traumatic stress disorder.

Perceived effectiveness of MC for both Veterans and non-Veterans was similar for the most common conditions and included: insomnia (8.3 vs. 8), PTSD (8.3 vs. 8.4), depression (8 vs. 8.2), anxiety (8 vs. 8.4), acute pain (7.8 vs. 7.8), chronic pain (7.5 vs. 7.8), and arthritis (7.4 vs. 8) (Table 3). Perceived effectiveness scores were similar for male and female Veterans and non-Veterans. The mean score for effectiveness across all conditions was 7.4 (SD = 1.1).

**Table 3.** Mean Scores of Perceived Effectiveness of Different Conditions

	Males (***N*** = 103)		Females (***N*** = 39)	
	Veterans (***N*** = 75)	Non-veterans (***N*** = 22)	Female veterans (***N*** = 15)	Female non-veterans (***N*** = 24)
Headaches/migraines	7.5	7	6.5	7.4
Arthritis	7.3	7.8	7.5	7.7
Chronic pain	7.7	8.1	6.7	7.5
Acute pain	8	8.5	6.5	6.8
Depression	8.1	7.3	7.8	8
Anxiety	8.1	9	7.8	7.7
Sleeping problems	8.2	7.7	8.7	7.7
PTSD	8.3	8.5	8.4	8

Data are presented as *n* (%) or mean (median).

The majority of respondents had used MC daily for at least 1 year, with oils identified as the most common mode of administration (58%; *n* = 91), followed by edibles (38%; *n* = 59) and vaporized cannabis (34%; *n* = 54) (Table 4). Both male and female Veterans were significantly more likely to use edible modes of administration compared with non-Veterans (65% vs. 9%, *p* < 0.001 and 67% vs. 25%, *p* < 0.01).

**Table 4.** Cannabis Use Patterns

	Males (***N*** = 103)	Females (***N*** = 39)		
	Veterans (***N*** = 75)	Non-veterans (***N*** = 22)	Female veterans (***N*** = 15)	Female non-veterans (***N*** = 24)
Average mg/day THC	19.5 (10)	20.4 (11.5)	14.7 (5)	10 (3)
Average g/day Herbal	3.5 (3)	1.7 (2)	2.6 (2.25)	1.2 (1.5)
Average mg/day: CBD	49.2 (10)	53.2 (30)	48.2 (10)	14.8 (6.5)
Frequency: More than once daily	45 (44%)	15 (68%)	5 (33%)	12 (50%)
Frequency: Daily or almost daily	21 (20%)	5 (23%)	4 (27%)	7 (29%)
Duration:1–2 years or less	31 (30	11 (50%)	7 (47%)	12 (50%)
Duration: More than 2 years	37 (36%)	9 (41%)	7 (47%)	10 (42%)
Mode of administration: Oil	36 (48%)	16 (73%)	7 (47%)	16 (66%)
Mode of administration: Edible	**49 (65%)** ^***^	**2 (9%)** ^***^	**10 (67%)** ^**^	**6 (25%)** ^**^
Mode of administration: Tincture	6 (8%)	1 (5%)	0	1 (4%)
Mode of administration: Smoked	24 (32%)	2 (9%)	2 (13%)	3 (12%)
Mode of administration: Vaporized	35 (47%)	7 (32%)	4 (27%)	4 (17%)
Mode of administration: Topical	9 (12%)	2 (9%)	2 (13%)	1 (4%)
Preferred strain: Indica	28 (37%)	4 (18%)	6 (40%)	3 (12%)
Preferred strain: Sativa	22 (29%)	1 (5%)	4 (27%)	2 (8%)
No. of different conditions				
1	2	4	1	3
2	6	2	0	7
3	10	5	2	3
4	11	3	2	3
5 and more	**39** ^***^	**4** ^***^	10	6

The bold values represent all the values that are significantly different.

Data are presented as *n* (%) or mean (median).

^**^ *p* < 0.01, ^***^p < 0.001.

CBD, cannabidiol; THC, delta-9 tetrahydrocannabinol.

Although not statistically significant, males tended to use MC in higher doses as compared with females (median amount for THC: 11.5 mg/day vs. 4 mg/day; and median amount for CBD 20 mg/day vs. 8.5 mg/day). Male Veterans were more likely to use MC to treat a greater number of conditions, including arthritis and several mental health problems (*p* < 0.01).

**FIG. 1.**
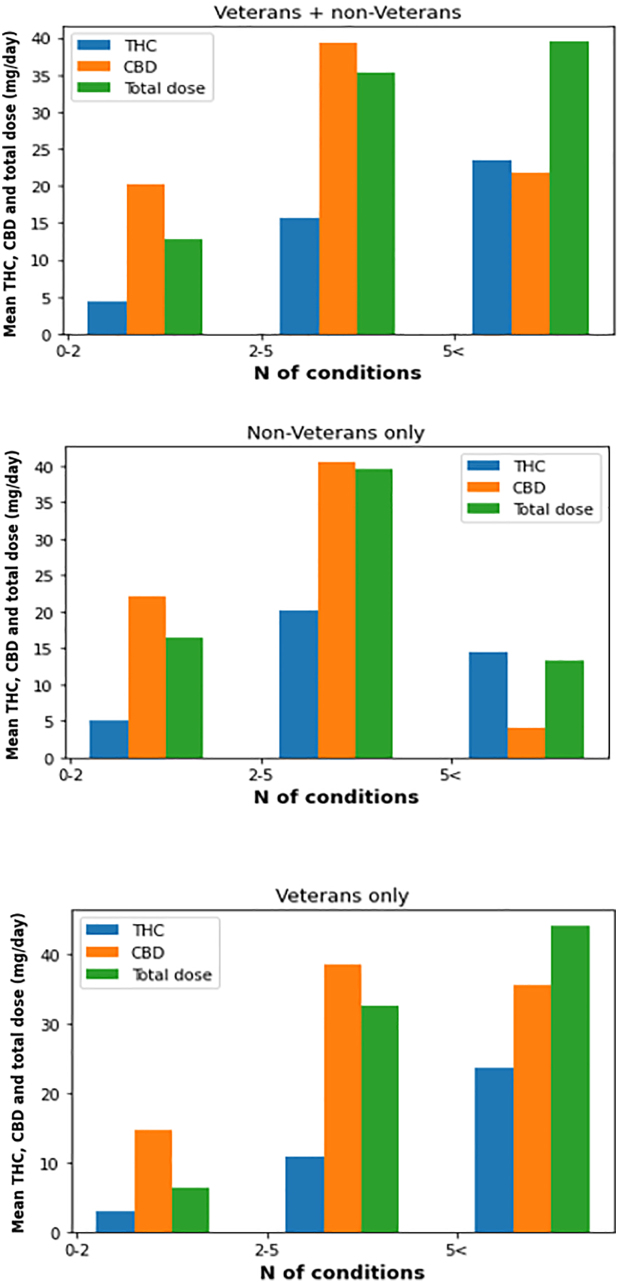
The association between number of conditions that MC is used for, and CBD, THC, and total doses. CBD, cannabidiol; MC, medical cannabis; THC, delta-9 tetrahydrocannabinol.

The dose of THC was positively associated with the number of conditions being treated (*p* = 0.01), whereas there was no statistically significant association between number of conditions and CBD doses (*p* = 0.8) (Fig. 1).

In multivariable analyses, adjusting for age and sex, Veterans compared with non-Veterans were more likely to use MC for depression, anxiety, PTSD, sleeping problems, and arthritis and were more likely to use an edible mode of administration (all *p*-values <0.001).

Veterans were significantly less likely than non-Veterans to be concerned about the safety and adverse effects of MC use (*p* = 0.017). One hundred and eleven (75%) respondents from both groups reported that their MC use was never out of control, and 95 (64%) respondents indicated that they never wished to stop the use of MC.

## Discussion

The results of this study indicate many similarities between MC use by male and female Veterans and non-Veterans. Daily amounts of ingested THC, CBD, and the herbal product were similar for Veterans and non-Veterans. Both groups used MC to treat a variety of ailments and reported that MC provided substantial relief for both physical and mental health problems.

The main difference in MC choice was that edibles were more commonly used by Veterans. Reasons for greater use of edibles could be due to the longer duration of action of the ingested product compared with vaporized forms. Stigma associated with inhalation, with the tendency to associate inhaled cannabis with recreational use may also have played a role in the preferential use of orally administered products. Oral administration can also be more discrete as there is avoidance of the smell associated with the inhaled product.

Alternately, cost issues may have influenced the selection of preferred method of administration as MC products are fully reimbursed for Veterans compared with the out-of-pocket expenses for other Canadians. It is possible that the non-Veterans using MC may have accessed the product at times via the less expensive illegal market that focuses mostly on the dried product that is most conveniently inhaled.

Differences between Veterans and non-Veterans regarding MC usage may also arise due to the fact that Veterans have unique health care needs resulting from their military service, including physical injuries, post-traumatic stress disorder (PTSD), traumatic brain injuries, or other service-related conditions.^1,2^

These specific conditions could potentially influence the reasons for seeking MC treatment and the effectiveness of its use compared with non-Veterans. In addition, there might be variations in the availability and quality of research, as well as clinical guidelines, for MC usage among Veterans compared with the general population. The unique circumstances and needs of Veterans could influence the focus and direction of research efforts, leading to potentially different recommendations and guidelines specific to this population. It is important to note that the extent of these differences may vary and are subject to further investigation and research.

Consistent with previous studies, these results indicate that MC is commonly used for a variety of physical and mental health complaints such as chronic pain, insomnia, anxiety, depression, PTSD, and arthritis.^14,15^ This prevalent use of and satisfaction with MC, often by self-administration, is in contrast to the limited evidence for substantial beneficial effects in the published literature.^4,16,17^

Despite the lack of strong scientific evidence demonstrating the effectiveness of MC compared with placebo, there was a strong consistent belief among participants that MC was effective for treating a wide range of physical and mental health conditions. Perceived effectiveness scores were particularly high for mental health problems, with average scores of 8 or greater.

These results are consistent with previous observational studies.^18–22^ Whether this represents a placebo effect, regression to the mean, or true efficacy will require more scientific study. These data do provide guidance in targeting these studies to the management of chronic pain conditions and several mental health problems.

On average, MC was used by most respondents several times a day, which was in line with other studies suggesting that the MC provided symptom relief rather than modifying the disease pathophysiology.^10,18,21,23,24^

A concerning observation of this study is that 60% of the respondents did not identify the strength or content (THC or CBD) of the cannabis product used. This lack of knowledge of product content by many users has been noted in previous studies.^25–27^ Although MC is reported to be used as a medicinal product, the poor attention to amount or content of the product may be bolstered by a perception of safety of cannabis as a recreational and natural product.

Alternately, lack of adequate knowledge by both patients and MC prescribers may also be a factor in poor knowledge of content. Further, we have observed limited concerns about safety and side effects of MC, especially from Veterans.

Consistent with other studies, one of the attractions of MC may be that it is used to treat multiple physical and mental health complaints.^18,20–22,28^ These multiple conditions are more prevalent among Canadian Veterans compared with the non-Veteran populations.

This may help with mitigating polypharmacy burden as well as dealing with adherence to complicated pharmaceutical routines and their side effects. However, the complexity of effects of the large numbers of cannabinoid and non-cannabinoid molecules in the plant *Cannabis sativa* requires both study and understanding before any evidence-based recommendation can be made in this regard.

Sex-based analyses are an important strength of this study. Although sex-based analyses are generally recognized as important tools for detecting disparities and providing insights for research and policy, this strategy has not always been used in research of Veteran populations. We did not observe any significant differences between males and females regarding their MC use patterns. Although males tend to use MC in greater doses, these differences were not statistically significant (*p* = 0.09).

Considering that females make up roughly 14% of the ∼600,000 Veterans now residing in Canada,^29^ female Veterans are grossly underrepresented in the literature with many studies reporting on male samples of Veterans (around 70% males).^30^ There is, thus, an urgent need to address the health-related needs of female Veterans in all future studies.

There are limitations to the current study that must be addressed. First, this was a convenience sample of respondents and the representativeness of these results to the population of users of MC remains to be confirmed. The response rate for this survey remains unknown as recruitment through links on social media or notices sent out by Veterans Affairs Canada does not provide an accurate denominator on how many MC users might have noticed the survey.

Second, the sample size was modest, which may reduce the accuracy of the estimates provided. Third, it is possible that this sample of MC users was biased toward those who found it effective, whereas those who did not benefit did not reply to the survey.

There remain many uncertainties around MC use, particularly among Veterans. Further study should address the specific molecular effects of herbal cannabis products, including various concentrations of THC and CBD, the contribution of other molecules such as terpenes and flavonoids to a therapeutic effect (termed the entourage effect), the interaction of cannabinoids with other medications, and the development of tolerance and importantly, adverse effects especially for long-term use. Ideal dosing as well as methods of administration of MC will also require attention.

## Conclusions

The responses from this sample of Canadians, both Veterans and non-Veterans, indicate the belief that MC is an effective therapy for both physical and mental health symptoms. While we identified some important differences in user characteristics and MC use patterns between Canadian Veterans and non-Veterans, daily dosage and the perceived effectiveness were similar.

These preliminary results should be considered when developing additional studies on MC use and effectiveness. Larger studies are required to validate these findings, but this study suggests that orally administered cannabis products for the primary conditions identified in this sample may be worth further study.

## Supplementary Material

Supplemental data

Supplemental data

## Supplementary Material

Supplementary Appendix SA1

CHERRIES reporting results.docx

## Abbreviations Used

CBD: cannabidiol
CUD: cannabis use disorder
MC: medical cannabis
MUHC: McGill University Health Centre
PTSD: post-traumatic stress disorder
THC: delta-9 tetrahydrocannabinol
