# Supplementary material for: Medical Cannabis Use Among Canadian Veterans and Non-Veterans: A National Survey
Source: Integr Med Rep. 2023 Oct 12;2(1):120–8. doi: 10.1089/imr.2023.0022 (PMC10619467; doi:10.1089/imr.2023.0022)
Supplement: Supplemental data [file Suppl_AppendixSA1.docx]

**Appendix 1** *(The survey questions):*

**1. During the past month have you used cannabis for medical reasons (to treat disease or improve symptoms)?**

- Yes
- No, but I am thinking about using Cannabis for medical reasons in the next month
- No
- No answer

**2. I currently use MC for the following conditions:**

- Epilepsy
- Multiple sclerosis, amyotrophic lateral sclerosis, spinal cord injury
- Arthritis
- Dystonia
- Huntington’s disease
- Parkinson’s disease
- Tourette’s syndrome
- Glaucoma
- Anxiety or stress
- Depression
- PTSD
- Schizophrenia/psychosis
- Alzheimer’s disease/dementia
- Skin diseases
- Irritable bowel syndrome
- Inflammatory bowel diseases (e.g., Crohn’s, colitis)
- Liver disease
- Obesity/diabetes
- Cancer
- Prefer not to say
- Other

**3. For which of the following symptoms do you use cannabis for medical purposes?**

- Acute pain (severe or sudden pain that resolves within a certain amount of time)
- Chronic non-cancer pain (persistent pain, lasting for months or even longer)
- Cancer pain
- Nausea/vomiting
- Wasting/weight loss and/or lack of appetite (e.g., from AIDS or cancer)
- Headaches/migraines
- Muscle spasms
- Seizures
- Problems sleeping
- Alcohol withdrawal symptoms
- Opioid withdrawal symptoms
- Palliative care
- Prefer not to say
- Other

**4. How much does cannabis help you with your disease/symptoms?['Condition']**

- From 1(not at all) to 10(Very much)

**5. Daily amount used currently (choose what applies):[ Herbal cannabis, THC g/day, CBD g/day]**

Open answer:

**6. Frequency of use:**

- More than once daily
- Daily or almost daily
- 3-4 days per week
- 1-2 days / week
- 2-3 days / month
- 1 day per month
- Less than 1 day per month
- No answer

**7. Mode of administration:**

- Edible
- Oil
- Tincture
- Smoked
- Vaporized
- Topical
- Other

**8. What is the THC /CBD product name?**

Open answer

**9. How long have you used MC?**

- < 1 year (91)
- 1 - 2 years (92)
- 3 - 4 years (93)
- 5 - 10 years (94)
- 10 years (95)
- No answer

**10. What is your preferred strength of THC / CBD?**

Open answer

**11. What is your preferred strain?**

- Indica (111)
- Sativa (112)
- Hybrid (113)
- No preference (114)
- No answer

**12. How concerned are you about the safety and side effects of MC use?**

- Very much concerned (141)
- Slightly concerned (142)
- Neither concerned nor unconcerned (143)
- Slightly not concerned (144)
- Very much unconcerned (145)
- No answer

**13. Did you think your use of cannabis was out of control?**

- Never / Almost never (000)
- Sometimes (001)
- Often (002)
- Always / Nearly always (003)
- No answer

**14. Did the prospect of missing a dose of cannabis makes you anxious or worried?**

- Never / Almost never (000)
- Sometimes (001)
- Often (002)
- Always / Nearly always (003)
- No answer

**15. Did you worry about your use of cannabis?**

- Never / Almost never (000)
- Sometimes (001)
- Often (002)
- Always / Nearly always (003)
- No answer

**16. Did you wish you could stop the use of cannabis?**

- Never / Almost never (000)
- Sometimes (001)
- Often (002)
- Always / Nearly always (003)
- No answer

**17. How difficult did you find it to stop, or go without cannabis?**

- Not difficult
- Quite difficult
- Very difficult
- Impossible
- No answer

**18. During the past 30 days, how often did you smoke cigarettes?**

- Daily
- Less than daily, but at least once a week
- Less than once a week, but at least once in the past month
- Not at all
- No answer

**19. If you smoke daily, on average how many cigarettes do you smoke each day?**

Open answer

**20. During the past 30 days, how often did you smoke tobacco in other forms (pipe, cigar, etc.)?**

- Daily
- Less than daily, but at least once a week
- Less than once a week, but at least once in the past month
- Not at all

**21. If you smoke tobacco in other forms daily, on average how many times per day do you smoke?**

Open answer

**22. How often do you have a drink containing alcohol?**

- Never
- Monthly or less
- 2 to 4 times a month
- 2 - 3 times a week
- 4 - 6 times a week
- Everyday
- No answer

**23. How many drinks containing alcohol do you have on a typical day when you are drinking?**

- 1-2 (261)
- 3-4 (262)
- 5-6 (263)
- 7-9 (264)
- 10 or more (265)
- No answer

**24. How would you currently rate your general health?**

- Excellent (271)
- Very good (272)
- Good (273)
- Fair (274)
- Poor (275)
- No answer

**25. Have you ever been treated for a substance abuse problem?**

- Answer
- Yes (1)
- No (0)
- Unknown / Prefer not to answer (3)
- No answer

**26. If you have been treated for a substance abuse problem, please describe.**

Open answer

**27. Over the past 2 weeks, how often have you been bothered by any of the following problems?**

- Answer
- Not at all (301)
- Several days (302)
- More than half the days (303)
- Nearly every day (304)
- No answer

**28. Over the past 2 weeks, how often have you been bothered by any of the following problems? (feeling down, feeling nervous, depressed, hopeless, anxious or on a edge)**

- Answer
- Not at all (301)
- Several days (302)
- More than half the days (303)
- Nearly every day (304)
- No answer

**29. Do you have pain that is always present?**

- Yes
- No
- No answer

**30. Do you have periods of pain that reoccur from time to time?**

- Yes
- No
- No answer

**31. How often does this pain limit your daily activities?**

- Answer
- Never (11)
- Rarely (12)
- Sometimes (13)
- Often (14)
- Always (15)
- No answer

**32. When you are experiencing this pain, how much difficulty do you have with your daily activities?**

- Answer
- No difficulty (21)
- Some difficulty (22)
- A lot of difficulty (23)
- You cannot do most activities (24)
- No answer

**33. How often do you use massage therapy (self-massage or provided by partner) to reduce your symptoms?**

- Answer
- More than once a day (421)
- Every day (422)
- 5-7 days per week (423)
- 3-4 days per week (424)
- 1-2 days per week (425)
- Less than once per week (426)
- Occasionally (427)
- Rarely (428)
- Never (429)
- No answer

**34. How often does the massage therapy improve your symptoms?**

- Answer
- All the time (431)
- Some of the time (432)
- Rarely (433)
- Never (434)
- No answer

**35. During the past 7 days, how much total time did you spend walking (at least 10 minutes at a time)?**

Open answer

**36. During the past 7 days, how much total time did you spend walking (at least 10 minutes at a time)?**

Open answer

**37. Not counting any time you answered for walking in the previous question, how much total time did you spend doing moderate physical activity (at least 10 minutes at a time)? Examples of moderate physical activity include carrying light loads, bicycling or swimming at a regular pace, doubles tennis, raking or picking up leaves, or sweeping floors.**

Open answer

**38. During the past 7 days, how much total time did you spend doing vigorous physical activity (at least 10 minutes at a time)? Examples of vigorous physical activity include aerobics, fast bicycling or swimming, jogging, playing soccer, heavy lifting, or digging. If you do no vigorous activity enter '0'.**

Open answer

**39. Rate how confident you are that you could perform the following activities. [I can walk briskly for 20 minutes without stopping.] [I can run or jog for 10 minutes without stopping.] [I can climb 3 flights of stairs without stopping.] [I can exercise for 20 minutes at a level hard enough to cause a large increase in heart rate and breathing.]**

- Answer
- Not at all Confident (391)
- Slightly Confident (392)
- Moderately Confident (393)
- Very Confident (394)
- Extremely Confident (395)
- No answer

**40. If we offered you a free web-based program to help you, your family, and friends improve their healthy lifestyle habits would you sign up?**

- Answer
- Yes (1)
- No (0)
- Don't know (3)
- No answer

**41. In your own words, what features would you like to see on the website?**

Open answer

**42. How important do you think each of the following factors are in determining whether you would take part in an on-line program specifically designed to help women and men improve their healthy lifestyle habits? [Knowing the on-line program was developed by experts in the field], [The on-line program was recommended by another veteran] [The program sends out e-mail reminders to re-visit the website] [The program sends out weekly tips to keep me motivated by e-mail] [The on-line program provides interactive features (e.g., taking quizzes and playing games)] [Being able to see my progress in reaching goals] [A family member or friends can sign up too] [The on-line program includes a feature to set personal goals] [The on-line program includes an on-line community for feedback and support] [The on-line program gives rewards (e.g., medallions or stars) when goals are achieved]**

- Answer
- Not at all important (411)
- Slightly Important (412)
- Moderately Important (413)
- Very Important (414)
- Extremely Important (415)
- No answer

**43. How old are you?**

Open answer

**44. What sex were you assigned at birth, meaning on your original birth certificate?**

- Male
- Female
- No answer

**45. What gender do you currently identify with?**

- Man
- Woman
- Other gender identity
- No answer

**46. What is your current weight? [kg] [lbs]**

Open answer

**47. How tall are you? [feets][inches][cm]**

Open answer

**48. I am a**

- Veteran
- Family member of Veteran
- Caregiver for a veteran
- Other
- No answer

**49. Were you born in Canada?**

- Yes (1)
- No (0)
- No answer

**50. In what region were you born?**

Open answer

**51. How many years have you lived in Canada?**

Open answer

**52. Which group best represents your ethnic origin?**

- Aboriginal (e.g., First Nations, Inuit, Métis) (531)
- Arab (532)
- Black (533)
- Chinese (534)
- Filipino (535)
- Japanese (536)
- Korean (537)
- Latin American (538)
- South Asian (e.g. Indian, Pakistani, Bangladeshi, Sri Lankan) (539)
- Southeast Asian (e.g. Cambodian, Indonesian, Laotian, Vietnamese) (540)
- West Asian (e.g. Afghan, Iranian) (541)
- Caucaisian / White (542)
- Other
- No answer

**53. I am**

- Employed (541)
- Retired (542)
- Unemployed (543)
- Student (544)
- Other
- No answer

**54. Which of the following best describes the area where you live?**

- Urban (551)
- Suburban (552)
- Rural (553)
- No answer

**55. Marital Status – Which of the following best describes you now?**

- Single (561)
- Divorced / Separated (562)
- Married / Co-habitating (563)
- Widowed (564)
- No answer

**57. Do you have dependents living at home (children or older relatives that you care for)?**

- Yes (1)
- No (0)
- No answer

**58. What is the highest level of education that you completed?**

- Less than high school (581)
- Completed some high school (582)
- High school graduate or equivalent (583)
- Technical college, community college or CEGEP (584)
- Completed some university, but no degree (585)
- University graduate (586)
- Completed some post-graduate but no degree (587)
- Completed post-graduate school (e.g., MSc., M.D., PhD) (588)
- No answer
